# Ebola virus and Sudan virus infection in humans: a comparison to inform vaccine research and development

**DOI:** 10.1080/22221751.2026.2686465

**Published:** 2026-07-10

**Authors:** Hilary S. Whitworth, Gabriella Quintard, Victoria Oyeneye, Thomas S. Postler, Arianna Marini, Marija Zaric, Patricia Fast, Margaret Meller, Samuel Gurrion, Gaudensia Mutua, Nina Malkevich, Jon Heinrichs, Swati B. Gupta, Suzanna C. Francis

**Affiliations:** a Epidemiology, IAVI SA NPC, Cape Town, South Africa; b Epidemiology, IAVI Inc, New York, United States; c Emerging Infectious Diseases, IAVI Inc, New York, United States; d IAVI Vaccine Design and Development Lab, IAVI Inc, Jersey City, United States; e Global Clinical Immunology, IAVI Inc, London, UK; f Clinical Development, IAVI Inc, New York, United States; g Project Management, IAVI Inc, New York, United States; h IAVI Africa CLG, Nairobi, Kenya; i Discovery Science, IAVI Inc, New York, United States; j Epidemiology, IAVI Stichting, Amsterdam, The Netherlands

**Keywords:** Sudan virus, Ebola virus, epidemiology, natural history, immunopathology

## Abstract

Recent outbreaks of Ebola virus (EBOV) disease (EVD) and Sudan virus (SUDV) disease (SVD) in sub-Saharan Africa underscore the ongoing public health threat posed by orthoebolaviruses. While highly effective vaccines are licensed for prevention of EVD, these offer limited cross-protection against other orthoebolaviruses, and no vaccines are licensed for SVD. Candidate SUDV vaccines are in development, but the sporadic nature of SVD outbreaks poses challenges for demonstrating clinical efficacy. In this context, it is important to consider whether and how the extensive evidence generated for EBOV infection, disease, and vaccine development can be leveraged to support SUDV vaccine development, consistent with the WHO prototype pathogen approach. Here, we synthesize available human data on the epidemiology, natural history, pathogenesis, and immunology of EBOV and SUDV infection to delineate their key similarities and differences. EBOV has caused more outbreaks, cases, and deaths than SUDV and has been more geographically widespread. There may be some differences in the typical causes of outbreaks, although gaps remain in understanding animal reservoirs for both viruses. Despite more limited data for SUDV, available evidence indicates that EBOV and SUDV share similar routes and progression of infection in humans, with comparable pathology and clinical presentation. Case fatality rates (CFRs) are generally higher for EVD than SVD. However, CFRs are influenced by outbreak context and healthcare access and should not be interpreted in isolation as evidence of intrinsic viral virulence. Collectively, this synthesis supports continued advancement of SUDV vaccine development using EBOV-informed evidence, while integrating SUDV-specific data where available.

## Introduction

Ebola virus (EBOV; species *Orthoebolavirus zairense*, formerly *Zaire ebolavirus*) and Sudan virus (SUDV; species *Orthoebolavirus sudanense*, formerly *Sudan ebolavirus*) are among four orthoebolaviruses known to cause severe febrile disease in humans (Table 1), sometimes with hemorrhagic manifestations, and with exceptionally high fatality rates [1]. Both viruses are on the World Health Organization (WHO)’s recently updated list of pathogens that should be prioritized for research and development (R&D) of medical countermeasures [2]. Vaccines against EBOV disease (EVD) have already had demonstrable impact in curbing viral transmission and reducing morbidity and mortality from EVD during outbreaks in West and Central Africa, but there are currently no vaccines licensed for protection against SUDV disease (SVD). Several candidate SUDV vaccines exist [3, 4]; however, generation of clinical efficacy data to support applications for qualification or licensure remains a challenge. In an unprecedented achievement, a ring vaccination trial with a Vesicular Stomatitis Virus (VSV)-based candidate SUDV vaccine was implemented within just four days of announcement of the most recent SVD outbreak [5], yet the low case numbers limited the ability to evaluate vaccine protection. The unpredictable nature (i.e. occurrence, location, magnitude, and duration) of SVD outbreaks makes an adequately-powered, randomized, controlled efficacy trial largely infeasible, meaning that developers need to consider alternative ways to demonstrate vaccine protection and seek licensure to enable vaccine availability for the populations that need them [6, 7].

**Table 1.** **Previous and current nomenclature for orthoebolaviruses and orthomarburgviruses, correct at the time of writing.** This review focuses specifically on Ebola virus (EBOV; species *Orthoebolavirus zairense*) and Sudan virus (SUDV; species *Orthoebolavirus sudanense*).

Previous genus name	Current genus name	Previous species name	Current species name	Virus name	Disease
*Ebolavirus*	*Orthoebolavirus*	*Bombali ebolavirus*	*Orthoebolavirus bombaliense*	Bombali virus (BOMV)	NA^a^
		*Bundibugyo ebolavirus*	*Orthoebolavirus bundibugyoense*	Bundibugyo virus (BDBV)	BDBV disease (BVD)
		*Reston ebolavirus*	*Orthoebolavirus restonense*	Reston virus (RESTV)	NA^a^
		*Sudan ebolavirus*	*Orthoebolavirus sudanense*	Sudan virus (SUDV)	SUDV disease (SVD)
		*Taï Forest ebolavirus*	*Orthoebolavirus taiense*	Taï Forest virus (TAFV)	TAFV disease (TVD)
		*Zaire ebolavirus*	*Orthoebolavirus zairense*	Ebola virus (EBOV)	EBOV disease (EVD)
*Marburgvirus*	*Orthomarburgvirus*	*Marburg marburgvirus*	*Orthomarburgvirus marburgense*	Marburg virus (MARV)^b^	Marburg virus disease (MVD)^b^
				Ravn virus (RAVV)^b^	

^a^ Bombali virus and Reston virus are not known to be pathogenic in humans.

^b^ Marburg virus and Ravn virus are members of the same species. Disease caused by either of these two viruses is known as Marburg virus disease.

There has been substantial progress in EBOV vaccine R&D over the past decade, the large 2013–16 EVD epidemic (and the recognized shortcomings of the associated epidemic response) being a major catalyst. However, with the fewer SVD outbreaks and cases to date, progress in SUDV vaccine development has been slower [8, 9]. Therefore, it may be valuable to consider how experience and understanding of EBOV and EVD, and progress in EBOV vaccine R&D, can be leveraged to support SUDV vaccine development. This aligns with a novel approach promoted by the World Health Organization (WHO) and the Coalition for Epidemic Preparedness Innovations (CEPI), whereby “prototype pathogens’ are used as representative models for entire pathogen families, enabling broadly applicable tools and data to be leveraged and rapidly adapted for related threats [2, 10]. EBOV is the proposed “prototype pathogen” for the *Filoviridae* family [2].

In a recent review, we compared SVD and EVD in non-human primates (NHP), showing that experimental EBOV and SUDV infection result in broadly similar disease, characterized by a severe systemic inflammatory response and disseminated intravascular coagulopathy, leading to tissue and organ damage and fluid loss [11]. While NHP models provide a highly controlled framework for comparative assessments, differences between experimental infection and natural human disease are likely to exist, and the spectrum and variability of disease observed in outbreak settings may not be fully captured by high-dose challenge models. We recently reviewed and collated the available evidence on the epidemiology, natural history, pathogenesis, and immunology of SUDV infection and disease in humans, based on data from SVD outbreaks over the past 50 years [3]; here, we draw on that review, alongside the extensive evidence on EBOV and EVD (where possible leveraging existing high-quality reviews and systematic reviews), to compare the characteristics of SUDV and EBOV infections in humans.

## Note on filoviral nomenclature

A history of confusion surrounding filoviral nomenclature somewhat muddies the water in the available literature [12]. Terms referring to the collective members of the now-outdated genus *Ebolavirus* (i.e. “ebolaviral”, “ebolavirus’, “ebolaviruses’) have often been confused with EBOV, a specific member of that genus. Accordingly, there has been confusion between the terms Ebola disease – a non-specific term that does not, on its own, distinguish the causative orthoebolavirus – and Ebola virus disease (EVD), which refers specifically to disease caused by EBOV. A similar problem exists for the now-outdated genus *Marburgvirus*, and for Marburg virus and Marburg disease [12]. Further, earlier species names have often been incorrectly used as virus names. For example, EBOV and SUDV have been incorrectly referred to as Zaire ebolavirus and Sudan ebolavirus, respectively, perhaps in part due to the first-mentioned ambiguity.

In 2023, the International Committee on Taxonomy of Viruses (ICTV) approved a taxonomic proposal to remove ambiguity in filoviral nomenclature through renaming the genera *Ebolavirus* to *Orthoebolavirus* and *Marburgvirus* to *Orthomarburgvirus* [12]. This followed an earlier ratification of a new codified rule for the naming of species in general to enable clearer differentiation between the name of a virus and its species [12, 13]. Outdated and current nomenclature for orthoebolaviruses and orthomarburgviruses are shown in Table 1.

While these amendments are a welcome step to promote clarity in filoviral nomenclature, there remain several decades of published literature with prevalent examples of ambiguity, with implications for efforts to examine and distinguish specific viruses and their respective diseases. For this review, the primary challenge was disentanglement of data and narratives that describe EBOV and EVD specifically versus orthoebolaviruses and Ebola disease (which may include SVD) more generally. Publications and data were only included where they distinguished between viruses or species (even if using incorrect nomenclature), or where the relevant virus was apparent from reference to a particular outbreak location and year. Throughout the remainder of this paper, we use the term EVD to refer specifically to disease caused by EBOV, and we use the term SVD to refer specifically to disease caused by SUDV.

## Overview of human EVD and SVD outbreaks in sub-Saharan Africa to date

### Outbreak history

**Figure 1.**
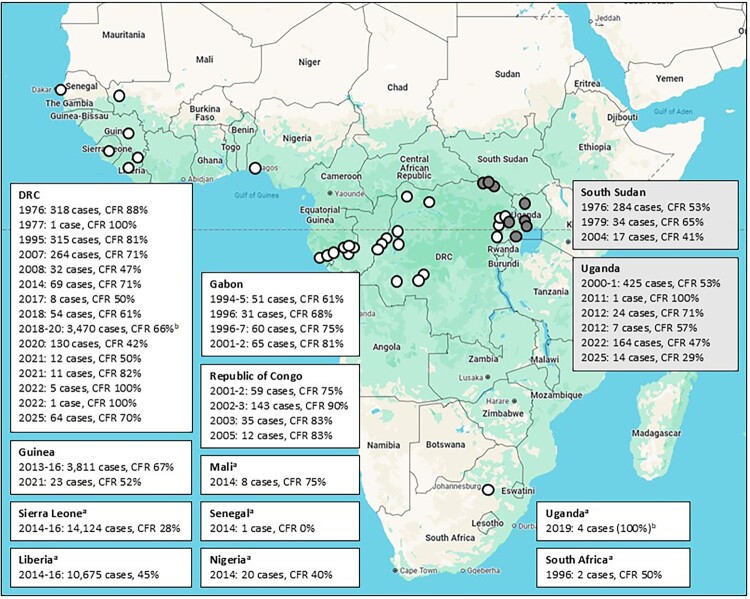
**History of reported EVD and SVD outbreaks in sub-Saharan Africa, by country.** EVD outbreaks are shown by white dots, and brief details (year of outbreak, number of reported cases, CFR) are provided in white boxes. SVD outbreaks are shown by grey dots, and brief details are provided in grey boxes. ^a^EVD outbreaks in these countries resulted from cross-border spread of infection from another affected country. ^b^Four EVD cases from 2019 were recorded in both the DRC and Uganda due to cross-border movement; all four cases acquired infection and died in the DRC. Abbreviations: CFR, case-fatality rate; DRC, Democratic Republic of Congo; EVD, Ebola virus disease; SVD, Sudan virus disease.

EBOV and SUDV were both discovered in 1976, when disease outbreaks emerged almost simultaneously in the Democratic Republic of the Congo (DRC, at the time known as Zaire) and in the Republic of South Sudan (at the time known as Southern Sudan, within Sudan) – though SVD was called “Ebola hemorrhagic fever” until after a later outbreak in 2000 [3, 14, 15]. The 1976 SVD outbreak occurred from June to November across four towns on the southern border of South Sudan and resulted in 284 cases and 151 deaths (case fatality rate [CFR] 53%) (Table 2; Figure 1) [3]. The EVD outbreak occurred from August to November in the Western Équateur province of the DRC and resulted in 318 cases and 280 deaths (CFR 88%) (Table 3; Figure 1) [14]. At the time, it was thought that the EVD outbreak in the DRC resulted from spread of infection from South Sudan; extensive traffic between the two areas was noted. However, an epidemiological link was never identified, and it was later established that the outbreaks were caused by viruses belonging to distinct species [16].

**Table 2.** Characteristics of SVD outbreaks reported in sub-Saharan Africa to date.

Year Location	**N cases**^a^	**N deaths CFR (95% CI)**^b^	Source	Index case	Human-to human transmission	**Outbreak duration**^c^	Geographic spread	Case demographics
1976 South Sudan^d^	284 total	151 53% (47-59%)	Spillover, bats suspected	Textile factory worker	Community, intrafamilial, nosocomial (incl. use of contaminated needles)	21wk	4 towns	NR
1979 South Sudan^d^	34 total 10 confirmed	22 65% (46-80%)	Spillover, bats suspected	Textile factory worker	Intrafamilial, nosocomial	10wk	2 towns	38% M Age range 10m-60y 6% <19y 6% HCW
2000-01 Uganda	425 total 218 confirmed	224 53% (48-58%)	Spillover	Farmer	Funeral, intrafamilial, nosocomial	24wk	3 districts	37% M Age range 3d-72y Median age 27y 7% HCW
2004 South Sudan^d^	17 total 13 confirmed	7 41% (18-67%)	Single spillover from NHP	Villager with recent hunting history	Intrafamilial, nosocomial	10wk	1 town	59% M Age range 6m-60y Median age 33y 6% <15y
2011 Uganda	1 confirmed	1 100% (3-100%)	Single spillover, bats suspected	Child from rural village	None	NA	-	F 12y
2012 Uganda	24 total 11 confirmed	17 71% (49-87%)	Spillover	Villager with recent history of agricultural work	Funeral, intrafamilial, nosocomial	5wk	1 town	NR
2012 Uganda	7 total 6 confirmed	4 57% (18-90%)	Spillover	Motorcycle taxi rider	Funeral, intrafamilial	6wk	1 town	NR
2022 Uganda	164 total 142 confirmed	77 47% (39-55%)	Spillover, bats suspected	Villager	Funeral, household, nosocomial, sexual, vertical	16wk	9 districts	58% M Median age 29y IQR 20-38y 13% HCW
2025 Uganda	14 total 12 confirmed	4 29% (8-58%)	Single spillover	HCW	Community, intrafamilial, nosocomial, vertical	5wk	6 districts	55% M Age range 20d-55y Mean age 27y 50% HCW

^a^ Total cases include laboratory confirmed and probable cases. Numbers of laboratory-confirmed cases are presented alongside total cases where available.

^b^ Exact (Clopper-Pearson) 95% CI were calculated, assuming a binomial distribution.

^c^ Approximate estimate of duration, including the likely first cases before the outbreak was confirmed, but excluding the 42-day countdown to declare the end of the outbreak.

^d^ At the time known as Southern Sudan, within Sudan. Abbreviations: CFR, case fatality rate; CI, confidence interval; d, days; F, female; HCW, healthcare workers; IQR, interquartile range; M, male; m, months; N, number; NA, not applicable; NHP, non-human primate(s); NR, not reported; SVD, Sudan virus disease; wk, weeks; y, years.

**Table 3.** **Characteristics of EVD outbreaks and epidemics reported in sub-Saharan Africa to date.** Epidemiologically linked outbreaks spanning multiple countries are grouped and summarized jointly.

Year, Location	**N cases**^a^	**N deaths CFR (95% CI)**^b^	Source	Index case(s)	Human-to human transmission	**Outbreak duration**^c^	Geographic spread	Case demographics
1976 DRC	318 total	280 88% (84-91%)	Spillover from NHP, antelope	Hospital patient	Community, household, nosocomial (incl. use of contaminated needles)	7wk	55 villages	44% M 20% <15y 5% HCW
1977 DRC	1 total	1 100% (3-100%)	Suspected resurgence from 1976 outbreak	Child	None	NA	NA	F 9y
1994-95 Gabon	51 total	31 61% (46-74%)	Spillover, NHP suspected	Gold Miners	Community, household, nosocomial	6wk	10 villages	NR
1995 DRC	315 total	254 81% (76-85%)	Spillover	Charcoal worker & farmer	Community, household, nosocomial	30wk	∼30 villages	20–25% HCW
1996 Gabon	31 total	21 68% (49-83%)	Spillover from NHP	Hunters	Household	12wk	2 villages	58% children
1996-97 Gabon & South Africa	62 total	46 74% (62-84%)	Spillover from NHP, importation	Hunter	Community, nosocomial	26wk	2 countries; 3 regions in Gabon	NR
2001-02 ROC & Gabon	124 total 37 confirmed	97 78% (70-85%)	Multiple spillovers from NHP, antelope, Importation	Hunters	Community, nosocomial	20wk	5 districts across 2 countries	50% M Age range 0-85y 27% <15y 2% HCW
2002-03 ROC	143 total	128 90% (83-94%)	Spillover from NHP, antelope	Hunters	Community, household	19wk	2 districts	53% M Age range: 5d – 80y
2003 ROC	35 total	29 83% (66-93%)	Spillover from NHP or boar	Hunters	Community, household	4wk	1 district	NR
2005 ROC	12 total	10 83% (52-98%)	Multiple spillovers from NHP, antelope	Poachers	Community, household	5wk	2 districts	83% M Age range 16-57y
2007 DRC	264 total	187 71% (65-76%)	Spillover, bats suspected	Village Chief & hunter	Community, nosocomial	9wk	2 health zones	NR
2008 DRC	32 total	15 47% (29-65%)	Suspected resurgence from 2007 outbreak	Postpartum mother	Community, nosocomial	11wk	1 district	NR
2013-16 W Africa	28,639 total 28,610 confirmed	11,308 39% (39-40%)	Spillover, bats suspected; Importation	Infant	Community, household, funeral, nosocomial transmission, lab-acquired	28m	6 countries in SSA, & 10 countries worldwide	5% HCW
2014 DRC	69 total 66 confirmed	49 71% (59-81%)	Spillover from NHP	Pregnant woman, married to bushmeat hunter	Funeral, community	12wk	4 districts	52% F Age range <5-60y 12% HCW
2017 DRC	8 total	4 50% (16-84%)	Single spillover from NHP, wild boar	Villager with recent history of butchering bushmeat	Community, nosocomial	3wk	7 towns	75% M Age range 16-60y 13% <18y
2018 DRC	54 total 38 confirmed	33 61% (47-74%)	Spillover	Police officer	Community, nosocomial	11wk	3 health zones	60% M Age range 8-80y Median 41y 21% HCWs
2018-20 DRC^b^	3,470 total 3,317 confirmed	2,287 66% (64-67%)	Spillover	Villagers with history of bush meat consumption	Community, household, nosocomial, funeral	23m	18 health zones	56% F Age range: 0-80y 29% <18 years 5% HCWs
2020 DRC	130 total 119 confirmed	55 42% (34-51%)	Spillover & suspected resurgence from 2018 outbreak	Woman with frequent bat consumption	Community, funeral, household, nosocomial	20wk	13 health zones	55% M Age range: <5-55+ 23% <18y 2% HCW
2021 DRC	12 total 11 confirmed	6 50% (21-79%)	Suspected resurgence from 2018-20 outbreak	Suspected 2018-2020 survivor	Household, nosocomial, possible sexual transmission	12wk	4 districts	17% HCW
2021 DRC	11 total 8 confirmed	9 82% (48-98%)	Suspected resurgence from 2018-20 outbreak	3-year-old child	Community, nosocomial	10wk	4 towns	50% <5y
2021 Guinea	23 total 16 confirmed	12 52% (31-73%)	Suspected resurgence from 2013-16 epidemic	HCW	Funeral, nosocomial	18wk	2 regions	22% HCW
2022 DRC	5 total 4 confirmed	5 100% (48-100%)	Spillover	Student	Community, intrafamilial	10wk	3 towns	80% M Age range 9-48y
2022 DRC	1 total 1 confirmed	1 100% (3-100%)	Suspected resurgence from 2018-20 outbreak	Hospitalized patient	None	NA	NA	F 46y
2025 DRC	64 total 53 confirmed	45 70% (58-81%)	Single spillover	Pregnant woman	Community, funeral, household, nosocomial, vertical	6wk	1 health zone	42% M Age range 0-65y 25% <9y 8% HCW

^a^ Total cases include laboratory confirmed and probable cases. Numbers of laboratory-confirmed cases are presented alongside total cases where available.

^b^ Exact (Clopper-Pearson) 95% CI were calculated, assuming a binomial distribution.

^c^ Approximate estimate of duration, including the likely first cases before the outbreak was confirmed, but excluding the 42-day countdown to declare the end of the outbreak.

^d^ Four EVD cases from the 2019 DRC outbreak were recorded in both DRC and Uganda due to cross-border movement; all four cases acquired infection and died in the DRC, so Uganda is omitted for the purpose of this table. Abbreviations: CFR, case fatality rate; CI, confidence interval; d, days; DRC, Democratic Republic of Congo; EVD, Ebola virus disease; F, female; HCW, healthcare workers; IQR, interquartile range; M, male; m, months; N, number; NA, not applicable; NHP, non-human primate(s); NR, not reported; ROC, Republic of Congo; SSA, Sub-Saharan Africa; W Africa, West Africa; wk, weeks; y, years.

**Figure 2.**
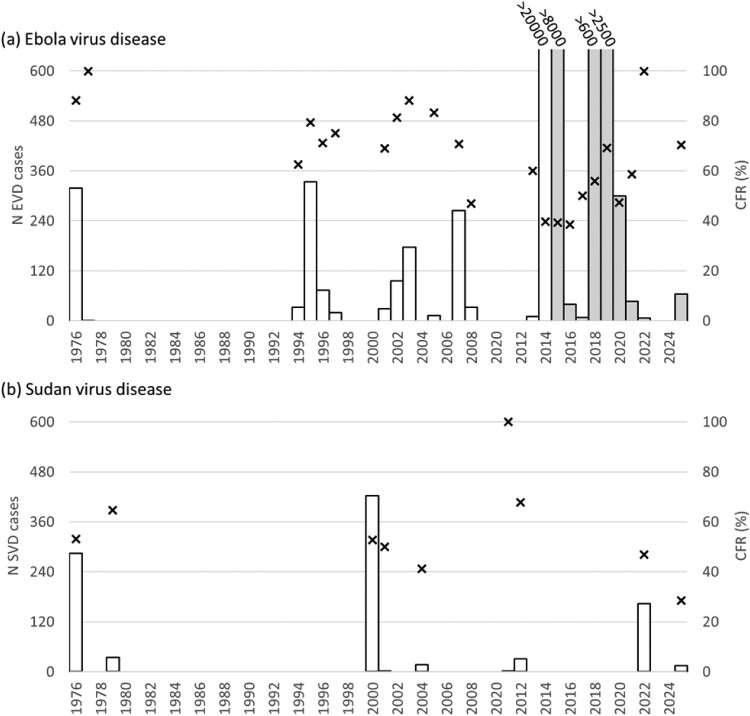
**Number of reported disease cases (bars) caused by (a) EBOV and (b) SUDV and associated CFRs (crosses) per year since 1976.** For multi-year outbreaks, annual distributions of cases and CFRs were approximated using data from periodic outbreak updates. In instances where multiple outbreaks occurred in the same year, figures reflect cumulative case counts and combined CFRs. Grey bars are shown for years when EBOV vaccines were available during EVD outbreaks, either in a trial setting or through wider vaccine deployment. Abbreviations: CFR, case-fatality rate; EBOV, Ebola virus; EVD, Ebola virus disease; N, number; SUDV, Sudan virus; SVD, Sudan virus disease.

Since then, EBOV has caused most reported filoviral disease outbreaks, cases and deaths, which have been widespread across Central and West Africa (Table 3; Figures 1 and 2) [15]. SVD outbreaks, in contrast, have been limited to South Sudan and Uganda (Table 2; Figure 1) [3]. The largest EVD outbreak occurred in West Africa from 2013 to 2016 and resulted in over 28,600 cases and 11,300 deaths (CFR 39%) [15]. The index case originated in Guinea. The greatest burden of disease was borne by Guinea, Sierra Leone, and Liberia, but cases were also reported in Mali, Senegal, Nigeria, Italy, Spain, the United Kingdom (UK), and the United States of America (USA), with domestic transmission events in Mali, Nigeria, Italy, and the USA. The largest SVD outbreak spread across three districts in Uganda from 2000 to 2001 and resulted in 425 cases and 224 deaths (CFR 53%) [3, 15]. The most recent SVD outbreak emerged in Uganda's capital city, Kampala, in January 2025, and resulted in 14 cases and four deaths (CFR 29%) [17]. The most recent EVD outbreak was declared in the DRC just a few months after the end of that SVD outbreak – in September 2025 – and resulted in 64 cases and 45 deaths (CFR 70%) [18].

Reported outbreaks of both diseases appear to be increasing in frequency, particularly for EVD, as illustrated in Figure 2. More than 40% of all recorded EVD outbreaks have occurred within the past decade. Over 90% of reported EVD cases and deaths have occurred in the past 20 years, largely driven by two exceptionally large events – the 2013–16 West Africa epidemic and the 2018–20 DRC outbreak. Similarly, approximately 80% of reported SVD outbreaks, and nearly 70% of reported SVD cases and deaths, have occurred since 2000.

### Outbreak sources

Most EVD outbreaks and all SVD outbreaks recorded to date arose from zoonotic spillover [3, 15, 19]. Bats may be a natural reservoir of both viruses, though this has never been confirmed [1, 3]. Other mammals, such as certain non-human primates (NHP; monkeys, gorillas, chimpanzees, baboons) and antelopes (duikers), can serve as intermediate hosts. Like humans, these animals often develop disease upon infection. For many EVD outbreaks, there is good epidemiological evidence for a transmission from an ill or deceased intermediate host, often linked to hunting, butchering, or consumption of bushmeat [19]. Conversely, an epidemiological link to infected NHP (baboon meat) has only been reported for one SVD outbreak to date [3, 19]. For several EVD and SVD outbreaks, transmission from bats was suspected but not confirmed. Major gaps remain in our understanding of animal reservoirs of infection and transmission sources for both viruses.

Notably, viral sequencing indicates that several recent EVD outbreaks resulted from viral reactivation in, or sexual transmission from, survivors from earlier outbreaks with persistent infection [15, 19, 20]. This has not been identified as a source of SVD outbreaks to date. However, post-convalescent persistence of SUDV in human semen and breast milk has been observed, and ocular and testicular SUDV persistence has been confirmed in NHP [3, 21].

### Phylogenetic analysis

While EBOV and SUDV are the most virulent of the orthoebolaviruses, phylogenetic analyses of isolates to date have demonstrated that EBOV in fact clusters more closely with Taï Forest virus (TAFV) and Bundibugyo virus (BDBV) [22]. Conversely, SUDV clusters more closely with Reston virus (RESTV), for which there is some evidence of infectivity but no evidence of pathogenicity in humans [22, 23]. An analysis of almost 200 orthoebolavirus genomes from the Virus Pathogen Resource (including 156 EBOV and 13 SUDV genomes) highlighted limited intra-species but greater inter-species variation [24]. Comparison of EBOV and SUDV specifically revealed 56-75% amino-acid sequence identity across the encoded proteins, with the greatest divergence observed in the surface glycoprotein (GP). Importantly, direct links between specific GP sequence features and characteristics of human disease remain poorly defined, particularly for SUDV.

## Clinical presentation and disease outcomes

A wealth of literature has been published describing the course of EBOV infection and EVD in humans, largely based on studies and observations from the 2013–16 EVD epidemic [25–27]. Data are more limited for SVD; the most comprehensive description of disease was constructed from observations during the outbreak in South Sudan in 1976, with accounts since then largely based on case reports or small case series [3]. Nonetheless, the available data demonstrate that EBOV and SUDV cause a similar severe and complex multisystem disease in humans, which can be rapid in onset and progression and often fatal [1, 3, 25–27]. The overall clinical picture broadly aligns with observations from experimentally infected NHP, where both diseases are characterized by fever, dehydration, coagulation abnormalities, multi-organ failure, and death [11].

### Incubation period

Infection with either virus can result in symptom onset in <48 h, but incubation periods of several weeks have been described [3, 28]. A systematic review of Ebola mathematical models and epidemiological parameters found that central estimates of the incubation period ranged from 1.7–29.6 days [28]. While estimates were derived from studies spanning the orthoebolaviruses (published up to 2023), most (37 of 43 studies) were of EBOV, and only three were of SUDV. The pooled mean incubation period estimate, based on nine studies, was 8.4 days (95% CI 8.0–8.8 days). There was some evidence for a difference across viral species, with a pooled mean of 8.4 days (95% CI 7.2–9.5 days) for EBOV and 4.8 days (95% CI 2.6–7.0) for SUDV, albeit with only two studies contributing data for SUDV. Notably, difficulties in confirming infection dates and the potential for transmission from undiagnosed intermediate cases present challenges to reliably estimating incubation periods. The best available estimate for SUDV likely comes from a subsequent publication describing a detailed epidemiological investigation of the 2022 SVD outbreak in Uganda. Based on 63 cases with clear exposure and onset dates, that study estimated a median incubation period of six days (interquartile range 5–8 days) [3]. For both viruses, shorter incubation periods are probably associated with higher infective doses [3, 29].

### Disease progression

Upon symptom onset, both diseases typically start with a non-specific febrile illness (including, for example, headache, fatigue, weakness, malaise, myalgia, arthralgia, and loss of appetite), which can increase in severity over several days [1, 3, 25–27]. For SVD, this has been described as an “influenza-like” illness, with cough and chest pain frequently reported [3]. In both diseases, these non-specific symptoms are often followed by severe gastrointestinal manifestations, including diarrhea, vomiting, abdominal pain, and anorexia, contributing to dehydration and weight loss [1, 3, 25–27]. Severe EVD or SVD can cause hemorrhagic manifestations such as epistaxis, hematuria, hematemesis, or conjunctival, gingival or vaginal bleeding, though the prevalence of these has varied considerably across outbreaks for both viruses [3, 30]. For SVD, the reported prevalence of abnormal bleeding was higher in earlier compared to more recent outbreaks [3]. For EVD, abnormal bleeding has generally been more prevalent in outbreaks in Central Africa compared to West Africa [25, 30]. A similar pattern has been observed for maculopapular rash [3, 26].

Neurologic and neuropsychiatric manifestations are seen with both EVD and SVD, and can range from mild (e.g. confusion) to severe (e.g. delirium with hallucinations, meningitis, encephalitis) [3, 31]. Breathing difficulties, throat pain, swallowing difficulties and hiccups are commonly reported in the acute stages of EVD, but have been less frequently reported for SVD [1, 3, 25–27]. Jaundice, cardiovascular abnormalities, and unconsciousness or coma have been occasionally reported for both viruses.

Notably, selection biases and reporting differences somewhat limit comparability of symptomatology across studies, outbreaks, and viruses. Thus, it is probably more useful to look at the bigger picture of disease and its progression than to focus on individual symptoms and their prevalence.

### Disease outcomes

Infection with either virus carries a high risk of death, typically occurring within two weeks of symptom onset and resulting from severe dehydration, hypovolemic or septic shock, and multi-organ failure [1, 3, 32]. CFRs are generally higher for EVD than SVD; based on data from outbreaks up to 2022, pooled CFRs for the two viruses are 67% (95% CI 56-77%) and 49% (95% CI 39-58%), respectively [33]. The CFR in the latest Ugandan SVD outbreak was lower, at 29% (95% CI 8-58%) [17]. CFRs are heavily influenced by access to, and uptake and quality of, medical services and treatment – which vary by country and setting. CFRs therefore reflect contextual determinants of disease outcomes in addition to any intrinsic differences in viral virulence. Accordingly, early supportive treatment, including fluid and electrolyte management, is critical for improving outcomes in both diseases. For both viruses, hemorrhagic manifestations are sometimes (though not always) predictive of a fatal outcome [3, 26, 34].

Survivors of either disease typically experience a slow and painful recovery, and long-term sequelae are common [3, 25, 35, 36]. The evidence base on long-term sequelae is more substantial for EVD, largely due to the extensive number (>17,000) of survivors from the 2013–16 EVD epidemic. Among EVD survivors, long-term pain, fatigue, hearing loss, visual impairment, and psychological disturbances are common [25, 35, 36]. The limited data on long-term sequelae following SVD indicate broadly similar manifestations, primarily musculoskeletal, neurological, ophthalmologic, and respiratory in nature [3].

### Possible asymptomatic or subclinical infection

For both EBOV and SUDV, there is some evidence of possible asymptomatic or mild infection, with virus-specific antibodies detected in case contacts who had no significant clinical manifestations [3, 37]. Due to the limitations of filoviral serological assays, the meaning of these findings is unclear [37]. Thus, major gaps remain in our understanding of the existence, burden, and implications of potential asymptomatic or mild EBOV and SUDV infection.

## Immunopathology

Historically, most insights into EBOV and SUDV immunopathology came from animal and *in vitro* studies. However, a large body of evidence accumulated during and following the 2000–01 SVD outbreak and 2013–16 EVD epidemic has improved understanding of human disease and allowed some comparison with animal models. While knowledge gaps remain, both EVD and SVD in humans appear to involve rapid viral replication, immune dysregulation with excessive inflammation, lymphopenia, endothelial dysfunction and vascular leakage, electrolyte and metabolic imbalance, and coagulopathy, leading to the often-catastrophic clinical picture described above [3, 25, 38, 39]. These features are broadly consistent with observations in experimentally infected NHP, where both infections result in high-level viremia, immune dysregulation with severe systemic inflammation and lymphopenia, and disseminated intravascular coagulopathy [11].

### Viral infection and replication

EBOV and SUDV infections typically begin through exposure of mucous membranes (or possibly skin), ingestion of viral particles, or inoculation via contaminated needles [1, 3, 39, 40]. Upon exposure, EBOV and SUDV infect susceptible host cells via GP binding, enabling viral replication [39, 41, 42]. Antigen-presenting cells (APCs) such as dendritic cells (DCs) and macrophages may be early, preferred targets [39]; productive infection of human DCs and macrophages by EBOV and SUDV has been demonstrated *in vitro* [43–45]. Viral dissemination may involve migration of infected DCs to lymph nodes, but is probably also driven by the cytokine microenvironment, recruiting immune cells – many of which may be infection targets – to sites of inflammation [3, 39, 46].

Viral RNA is generally detectable in blood from EVD and SVD patients from symptom onset, and RNA levels peak during acute infection [3, 47]. For both viruses, a steeper rise in viral RNA in early infection, and higher peak RNA levels, are consistently associated with more severe disease and a fatal outcome, likely indicating an inability of the host response to control viral replication and contain the infection [3, 47].

### Immune dysregulation with severe inflammation

Immune dysregulation and severe inflammation are widely considered to be hallmarks of EVD and SVD [3, 39]. Some evidence points towards early immune evasion by the viruses, possibly involving potent antagonism of host interferon (IFN) responses and contributing to pathogenesis through uncontrolled viral replication, dysregulated inflammation, and a delayed adaptive immune response [48, 49]. Infection with either virus typically causes a profound inflammatory response. Evidence consistently points towards massive upregulation and release of inflammatory mediators (cytokines, chemokines, free radicals, coagulation factors), correlating with viremia levels and clinical severity [3, 50–52]. Acute and persistent inflammation can cause abnormal, sustained activation of signalling pathways, potentially leading to tissue and organ damage [53]. In EVD and SVD, the inflammatory response intensifies as disease progresses and is associated with disease outcome, with uncontrolled cytokine release often observed in fatal disease [3, 46].

### Lymphopenia

Lymphopenia, possibly driven by a loss of peripheral T-cells or migration to infected tissues, has been reported in severe and fatal EVD and SVD [3, 46]. Although T-cells are not directly infected by either virus, the inflammatory environment may induce bystander cell death [39]. EBOV-infected PBMCs show massive T-cell and macrophage loss via apoptosis, and elevated circulating levels of inflammatory mediators known to be toxic to T-cells have been observed in severe or fatal SVD (e.g. nitric oxide [NO]) and EVD (e.g. tumor necrosis factor [TNF]) [3, 43, 46, 52]. Fatal EVD has also been associated with increased T-cell expression of inhibitory markers like PD-1, likely indicative of T-cell exhaustion and impaired functionality [54].

### Endothelial dysfunction and vascular leakage

Both experimental and clinical evidence suggest that endothelial dysfunction plays an important role in the pathogenesis of EVD and SVD, leading to vascular leakage and fluid extravasation [39, 55]. Dysfunction may arise due to direct viral effects, as well as indirectly from the excessive inflammatory response [55]. Inflammatory mediators such as TNF-α and NO – elevated levels of which have been observed in severe or fatal EVD and SVD – can increase endothelial activation and permeability [3, 52]. While vascular leakage supports immune cell trafficking, excessive extravasation can be harmful and manifest clinically as impaired perfusion, hypoalbuminemia, hemoconcentration, and hypovolemic shock – frequently reported features of severe EVD and SVD [3, 32]. Furthermore, endothelial activation may exacerbate inflammation and promote coagulopathy, also contributing to disease severity [39]. Elevated markers of endothelial activation or disruption (e.g. sICAM, thrombomodulin) have been repeatedly observed in severe SVD and EVD [3, 56].

### Electrolyte and metabolic imbalance

Numerous blood chemistry studies in EVD and SVD patients, together with insights from the substantial therapeutic effects of electrolyte and fluid administration, provide compelling evidence for a key role of electrolyte and metabolic imbalance in disease progression [32, 39, 57]. Low levels of blood calcium, sodium, and magnesium, together with potassium abnormalities, have been consistently observed in both EVD and SVD; use of clinical chemistry to monitor electrolyte balance and guide correction of abnormalities is a recognized approach for improved treatment [3, 58, 59]. Some electrolyte abnormalities probably occur secondary to gastrointestinal dysfunction, with profuse watery diarrhea being a common clinical feature of both diseases. Others may arise from infection of and damage to the kidneys, signalled by elevated levels of markers such as blood urea nitrogen (BUN) and creatinine in severe cases [3, 58, 59]. Untreated, electrolyte imbalances likely contribute to severe culminations of EVD and SVD such as cardiovascular abnormalities, seizures, or coma.

### Coagulopathy

Abnormal bleeding (often gastrointestinal, nasal, or conjunctival) occurs in some EVD and SVD patients. Reduced platelet counts or function, low levels of coagulation factors, and prolonged clotting times have been observed in both diseases, sometimes correlating with disease severity and outcome [3, 59, 60]. Studies have shown that D-dimer levels are elevated in EVD and SVD and associated with fatal SVD [3, 61]. This may be indicative of disseminated intravascular coagulation (DIC), though it is rare that sufficient laboratory data are available from patients to reliably evaluate whether DIC criteria are met.

Notably, liver damage – common in severe EVD and SVD and marked by elevated aspartate aminotransferase (AST), alanine transaminase (ALT), and bilirubin – may contribute to hemorrhagic manifestations through impaired coagulation factor synthesis [3, 39, 59]. Additionally, endothelial dysfunction (discussed above) may play a role in coagulopathy through the release of proteins such as thrombomodulin, tissue factor, and von Willebrand factor [62]. Upregulation and elevated levels of these markers have been associated with severe disease, hemorrhaging, or death in both SVD and EVD [3, 63].

### Severe and fatal disease

Vascular leakage, fluid loss from vomiting and diarrhea, and bleeding due to coagulopathy can all contribute to reduced blood volume in EVD and SVD, potentially leading to hypovolemic shock, tissue hypoperfusion, and multi-organ failure. Even with volume resuscitation, patients can still proceed to organ failure due to sepsis and the associated widespread inflammatory response. Disease outcome depends on both medical care and the host-virus interaction. Fatal cases are often linked to uncontrolled viral replication, excessive inflammation, and a suppressed adaptive response (discussed below) [3, 46, 47, 64]. While current evidence fails to pinpoint any consistent differences in the immunopathology of EVD versus SVD, knowledge gaps remain, and further research is warranted to better understand the possible contrast in virulence between the two viruses.

### Long term sequalae

Sustained immune activation and inflammation following convalescence have been proposed as contributors to long-term sequelae in survivors of EVD and SVD; prolonged immune dysregulation has been described following recovery from EVD [22, 65]. In addition, viral persistence in immune-privileged sites, including the eye, central nervous system, and reproductive organs, has been proposed as a potential mechanism underlying post-acute complications [3, 22, 66]. Evidence exists for EBOV persistence in multiple human tissues and fluids, including ocular fluid, cerebrospinal fluid, and semen [66]. For SUDV, persistence in semen has been documented in humans, and ocular and testicular persistence have been confirmed in NHP [3, 21]. However, direct causal links between viral persistence in immune-privileged sites and specific long-term clinical outcomes remain incompletely defined, and evidence for such associations is especially limited for SUDV.

## Adaptive immune response

Evaluations of host immune responses during infection and convalescence are limited for both viruses, particularly SUDV, due to the discernable challenges in conducting this type of research – for example, with infection risk and logistical challenges in collecting, transporting, storing, and testing samples, and the need to prioritize the outbreak response and patient care. However, studies in survivors have enabled evaluation of long-term responses. Correlates of protection are currently lacking for both viruses, but evidence suggests that a robust and sustained adaptive immune response is critical for survival [3, 64]. Observations from experimentally infected NHP are consistent with these findings, indicating disrupted antigen presentation and delayed or ineffective adaptive immune responses following EBOV and SUDV infection, although interpretation is limited by the predominance of fatal challenge models and the paucity of long-term survivor data in NHP [11].

### Humoral and cellular responses to infection

In EVD, EBOV-specific IgM is detectable from just two days after symptom onset, and IgG from 5–18 days [64]. An early and sustained IgG response is associated with EVD survival, while a weak IgM response and undetectable IgG are associated with death. EBOV-specific circulating T-cells have been observed in both EVD survivors and patients who succumbed to infection; however, the magnitude, diversity, and duration of those responses are typically greater among survivors [64, 67, 68]. Both CD4^+^ and CD8^+^ T-cell responses have been detected in systemic circulation, specific primarily to the viral nucleoprotein (NP), but also to the surface GP and other viral proteins. Fatal EVD is typically characterized by early T-cell activation and proliferation, followed by T-cell exhaustion and apoptosis, and an impaired adaptive response [64].

The evidence base for SVD is considerably more limited. One study suggested that efficient CD8^+^ T-cell activation and preservation of T-cell populations may be associated with SVD survival, while a suppressed response and loss of T cells may be predictive of a fatal outcome [3]. However, those evaluations were limited to general immunophenotyping and did not assess SUDV-specific responses. Similarly to EVD, a humoral immune response may be observed in SVD patients who survive, but typically undetectable in non-survivors [3].

### Long-term immune responses in survivors

For both EVD and SVD, there is evidence of long-lasting, multi-functional, virus-specific humoral and cellular responses post recovery in some survivors [3, 64]. Humoral responses exhibit both neutralizing activity and Fc-dependent effector functions. Long-lived cellular responses largely consist of polyfunctional CD4^+^ T cells expressing IFN-γ, IL-2, and TNF-α. Current evidence extends to 40 years post infection for EBOV, and 15 years for SUDV [3, 64].

## Transmissibility

Numerous robust epidemiological studies have examined human-to-human spread of EBOV and SUDV. Characteristics of spread within populations are similar for the two viruses [3, 69–71]. Viral persistence in survivors has transmission implications for both EBOV and SUDV [72, 73].

### Transmission routes

Human-to-human transmission of EBOV or SUDV occurs through close contact with a symptomatic case or their infectious body fluids. During outbreaks of either virus, most transmission has occurred in a healthcare or household setting, as well as among familial contacts outside the household [3, 40, 74]. The bodies of people who have succumbed to EVD or SVD remain infectious, and burials have presented as transmission hotspots when involving traditions of touching, washing, or kissing the deceased [3, 74]. Data from experimentally-infected NHP – intended to model human disease – suggest that fatal EVD cases may remain infectious for up to seven days post death [75]; data are lacking for SVD. Both viruses are spread through vertical or sexual transmission, but there is little or no evidence of natural aerosol, airborne, or fomite transmission [3, 40].

### Measures of transmissibility

Measures of transmissibility have varied considerably across outbreaks for both viruses. However, there is no clear evidence of a difference in the risk of transmission of EBOV versus SUDV. Basic reproduction number (R0) estimates have ranged from 0.05–12.0 for EBOV and 1.3–2.7 for SUDV, but pooled R0 estimates are very similar (EBOV: 1.9, 95% CI 1.7-2.2; SUDV: 2.0, 95% CI 1.3-2.8) [3, 28, 69]. Notably, most R0 estimates are derived from a small number of outbreaks (some with few cases) for both viruses [69].

R0 is a product of infectious period, contact rate, and transmission probability, and thus shaped by context and interventions. In contrast, the secondary attack rate (SAR) estimates the likelihood of transmission given a specific type of contact with an infectious case. The pooled household SAR for both EBOV and SUDV combined was estimated at 12.5% (95% CI 8.6-16.3%), with no evidence of a difference between the two viruses [70]. Super-spreading has been observed across multiple outbreaks for both EBOV and SUDV [3, 74, 76].

### Risk factors for transmission

The risk of human-to-human transmission of either EBOV or SUDV is considerably higher with direct compared to indirect contact [3, 71]. The household SAR for EBOV was estimated to be 29% among household members with direct contact, versus 0% in those with no direct contact [71]. Equivalent estimates for SUDV were 31-36% and 0-8%, respectively. Sleeping in the same room or bed has been identified as an important risk factor for transmission of both viruses [3, 71]. There is evidence that the risk of household transmission of SUDV is higher where the primary case is fatal [3]. This is probably also true for EBOV as severe and fatal cases have the greatest viral loads and produce larger volumes of infectious body fluids. For both viruses, transmission risk is highest with direct contact in a nursing or caring capacity (in the absence of appropriate infection control measures), particularly during the acute or late stages of disease [3, 71].

### Viral persistence

EBOV and SUDV can persist in immune-privileged sites in some survivors, with implications for onward viral transmission. Both viruses have been shown to remain detectable in semen following recovery – persisting for weeks, months or, in some cases, years – and sexual transmission from convalescent males has been reported [3, 72, 73]. For EBOV, re-emergence from persistently infected survivors is believed to have contributed both to flare-ups during the original outbreak and to the initiation of new outbreaks [15, 19, 20]. To date, there is no evidence of a new SVD outbreak arising from SUDV reactivation; however, documentation of long-term viral persistence in SVD survivors indicates that a theoretical risk may exist [3]. The absence of documented SVD outbreak reactivation thus far may reflect the substantially smaller number of SVD outbreaks and cases compared with EVD, rather than fundamental differences in viral persistence.

## Special populations and co-infections

Evidence on infection and disease in special populations is limited, particularly for SVD. However, both diseases appear to carry higher risks in children and pregnancy [3, 25, 77, 78]. Data on co-infections (e.g. SUDV-HIV, EBOV-malaria) are few and have methodological constraints that tend to limit inference [3, 25, 79].

### Children and pregnant women

Young children are often (though not always) at lower risk of acquiring EBOV and SUDV infection due to the type of contact that is usually associated with viral transmission between individuals (i.e. close and direct contact with an acutely symptomatic case, usually in a caregiving/nursing capacity) [3, 25, 78]. The most recent EVD outbreak in DRC is one of a few outliers, with a quarter of cases among children less than nine years old [18]. Nonetheless, upon infection with EBOV, children typically experience quicker disease onset and progression than adults (data are lacking for SUDV); and the risk of death appears to be higher in children than in adults for both viruses [3, 25, 78]. In the latest DRC outbreak, for example, the CFR was 70% overall but 92% among children aged 0–5 years [18]. Furthermore, both viruses can be transmitted from an infected mother to her infant, either *in utero* or possibly through breastfeeding [3, 40]. While early reports suggested that pregnancy might be associated with more severe EVD and higher case fatality, the data are mixed; a systematic found no clear evidence of a higher risk of EVD-associated death among pregnant compared to non-pregnant women, but the overall paucity of evidence limited definitive conclusions [77]. Similarly, data from SVD outbreaks to date do not support a difference in CFRs in pregnant and non-pregnant women, albeit based on small numbers of pregnant women. Nonetheless, infection with either virus almost always results in loss of the fetus or infant [3, 77].

### Co-infections

There is a paucity of research evaluating EBOV and SUDV infection and disease in people living with HIV (PLHIV). Although HIV infection has been suggested to increase susceptibility to EBOV infection and the risk of EVD-associated death due to underlying immunosuppression [80], robust human epidemiological data demonstrating increased EVD incidence or case fatality among PLHIV are lacking. The limited available data provide no evidence for an increased risk of SUDV infection, SVD, and SVD-associated death in PLHIV compared to people without HIV [3]. However, this is based on a single study with a small sample size, and the level of immunosuppression in the PLHIV was unknown (though anti-retroviral treatment was not widely available for HIV in Uganda at that time). There are conflicting data on whether malaria co-infection increases or decreases the risk of death from EVD, and data on malaria and SVD are lacking [25, 79].

## Summary and conclusions

**Figure 3.**
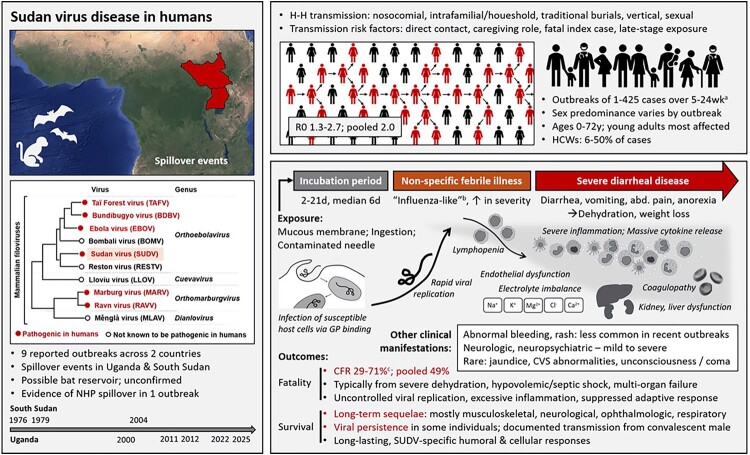
**Summary of Sudan virus disease (SVD) in humans.** The map in the left panel shows countries in which Sudan virus (SUDV) spillover events and associated SVD outbreaks have been reported. The timeline at the bottom of the same panel indicates the years in which SVD outbreaks were reported in South Sudan (formerly Southern Sudan) and Uganda; the outbreak that was reported in 2000 continued into 2001, and two outbreaks occurred in 2012. The phylogenetic tree was adapted from Groseth and Hoenen (NPJ Viruses, 2024). The graphic in the lower right panel depicts the current understanding of the routes and progression of SUDV infection and disease in humans. This is intended as an approximate representation and does not reflect exact timings, highlighting uncertainty in the existing evidence on human disease. ^a^Approximate estimates of duration, including the likely first cases before the outbreaks were confirmed, but excluding the 42-day countdown to declare the end of an outbreak. ^b^Including headache, fatigue, weakness, malaise, myalgia, arthralgia, anorexia, cough, and chest pain. ^c^Excluding the 2011 outbreak that consisted of just a single fatal case. Abbreviations: abd., abdominal; CFR, case fatality rate; CVS, cardiovascular system; d, days; GP, glycoprotein; HCWs, healthcare workers; H-H, human-to-human; NHP, non-human primates; R0, basic reproduction number; VHF, viral hemorrhagic fever; wk, weeks; y, years.

**Figure 4.**
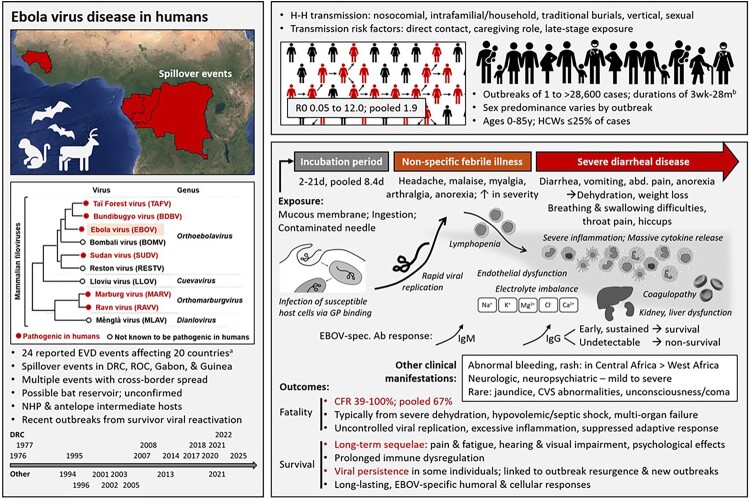
**Summary of Ebola virus disease (EVD) in humans.** The map in the left panel shows countries in which Ebola virus (EBOV) spillover events have been reported. Owing to cross-border spread during several outbreaks, the number of countries affected by EVD outbreaks is substantially larger than the number of primary spillover locations. The timeline at the bottom of the same panel indicates the years in which EVD outbreaks were reported in the Democratic Republic of the Congo (DRC), where most EVD outbreaks have occurred to date, and in other countries; some outbreaks spanned multiple years, and some years saw multiple outbreaks. The phylogenetic tree was adapted from Groseth and Hoenen (NPJ Viruses, 2024). The graphic in the lower right panel depicts the current understanding of the routes and progression of EBOV infection and disease in humans. This schematic is intended as an approximate representation and does not reflect exact timings, highlighting uncertainty in the existing evidence on human disease. ^a^The 24 events include epidemiologically linked outbreaks grouped across countries; disaggregation by country yields 32 outbreaks in SSA. The 20 affected countries include four in sub-Saharan Africa (SSA) with documented zoonotic spillover, seven additional SSA countries affected through cross-border transmission, and nine countries outside SSA affected via importation (including medical evacuation). ^b^Approximate estimates of duration, including the likely first cases before the outbreaks were confirmed, but excluding the 42-day countdown to declare the end of an outbreak. Abbreviations: Ab, antibody; abd., abdominal; CFR, case fatality rate; CVS, cardiovascular system; d, days; GP, glycoprotein; HCWs, healthcare workers; H-H, human-to-human; IgG, Immunoglobulin G; IgM, Immunoglobulin M; m, months; NHP, non-human primates; R0, basic reproduction number; ROC, Republic of Congo; VHF, viral hemorrhagic fever; wk, weeks; y, years.

This review provides a comprehensive comparison of SUDV and EBOV infection and disease in humans, with Figures 3 and 4 providing a visual synthesis of current understanding. To date, EBOV has caused more outbreaks, cases, and deaths, and EVD outbreaks have been more geographically widespread. There may be some differences in typical outbreak sources between EBOV and SUDV, though gaps remain in our understanding of animal reservoirs for both viruses. Several recent EVD outbreaks are believed to have arisen from long-lasting viral persistence and reactivation in survivors. While this has not been documented for SUDV to date, it is conceivable that a similar risk exists, particularly given evidence of viral persistence following SUDV infection.

Despite several gaps and limitations in the literature – particularly for SVD (discussed in detail previously [3]) – the available evidence suggests that EBOV and SUDV share similar routes and progression of infection in humans, and the resulting diseases are comparable in pathology and clinical presentation. These observations are consistent with findings from our earlier review comparing SUDV and EBOV infection and disease in experimentally infected NHP [11]. CFRs are generally higher for EVD than SVD. However, this is likely influenced by differences in outbreak location, setting, and timing, with implications for access to and quality of medical care. Thus, CFR estimates should not be interpreted as evidence of intrinsic differences in viral virulence in isolation. Notably, under comparable experimental conditions, NHP studies have not demonstrated consistent differences in CFRs between the two diseases, though these findings are derived from models using high infective doses [11]. For both viruses, an effective adaptive immune response appears important for survival in humans, although validated correlates of protection are currently lacking. Both EBOV and SUDV are highly contagious through close contact with acutely symptomatic cases, with similar risk factors for infection and little evidence for a meaningful difference in transmissibility. Survivors of both diseases may experience substantial long-term sequelae.

Taken together, delineation of the key similarities and differences between EVD and SVD provides a basis for understanding how the extensive evidence base generated for EBOV may be cautiously leveraged to support SUDV vaccine R&D, consistent with WHO and CEPI’s prototype pathogen approach [2, 10]. Similarities in human disease phenotype, immunopathology, and clinical course – supported by observations from experimentally infected NHP – may inform practical decisions around vaccine platforms, immunogen selection and dosing strategies, choice and interpretation of appropriate animal models, and the design of clinical studies, including adaptation of EBOV-informed clinical endpoints and case definitions for SUDV trials. Within this framework, and where vaccines are based on shared platforms, these comparisons may also support consideration of immunobridging approaches across viruses [6]. Collectively, this synthesis supports continued advancement of SUDV vaccine development using EBOV-informed evidence, while appropriately integrating SUDV-specific data where available.

## Supplementary Material

Tables and Figures_resubmission March 2026_cleaned.docx
