# Supplementary material for: Ebola virus and Sudan virus infection in humans: a comparison to inform vaccine research and development
Source: Emerg Microbes Infect. 2026 Jul 10;15(1):2686465. doi: 10.1080/22221751.2026.2686465 (PMC13360513; doi:10.1080/22221751.2026.2686465)
Supplement: Tables and Figures_resubmission March 2026_cleaned.docx [file TEMI_A_2686465_SM8477.docx]

**Table 1. Previous and current nomenclature for orthoebolaviruses and orthomarburgviruses, correct at the time of writing.** This review focuses specifically on Ebola virus (EBOV; species *Orthoebolavirus zairense*) and Sudan virus (SUDV; species *Orthoebolavirus sudanense*).

| **Previous genus name** | **Current genus name** | **Previous species name** | **Current species name** | **Virus name** | **Disease** |
| --- | --- | --- | --- | --- | --- |
| *Ebolavirus* | *Orthoebolavirus* | *Bombali ebolavirus* | *Orthoebolavirus bombaliense* | Bombali virus (BOMV) | NA^a^ |
|  |  | *Bundibugyo ebolavirus* | *Orthoebolavirus bundibugyoense* | Bundibugyo virus (BDBV) | BDBV disease (BVD) |
|  |  | *Reston ebolavirus* | *Orthoebolavirus restonense* | Reston virus (RESTV) | NA^a^ |
|  |  | *Sudan ebolavirus* | *Orthoebolavirus sudanense* | Sudan virus (SUDV) | SUDV disease (SVD) |
|  |  | *Taï Forest ebolavirus* | *Orthoebolavirus taiense* | Taï Forest virus (TAFV) | TAFV disease (TVD) |
|  |  | *Zaire ebolavirus* | *Orthoebolavirus zairense* | Ebola virus (EBOV) | EBOV disease (EVD) |
| *Marburgvirus* | *Orthomarburgvirus* | *Marburg marburgvirus* | *Orthomarburgvirus marburgense* | Marburg virus (MARV)^b^ | Marburg virus disease (MVD)^b^ |
|  |  |  |  | Ravn virus (RAVV)^b^ |  |

^a^Bombali virus and Reston virus are not known to be pathogenic in humans. ^b^Marburg virus and Ravn virus are members of the same species. Disease caused by either of these two viruses is known as Marburg virus disease.

**Table 2. Characteristics of SVD outbreaks reported in sub-Saharan Africa to date.**

| **Year**  **Location** | **N cases^a^** | **N deaths**  **CFR (95% CI)^b^** | **Source** | **Index case** | **Human-to human transmission** | **Outbreak duration^c^** | **Geographic spread** | **Case demographics** |
| --- | --- | --- | --- | --- | --- | --- | --- | --- |
| 1976  South Sudan^d^ | 284 total | 151  53% (47-59%) | Spillover, bats suspected | Textile factory worker | Community, intrafamilial, nosocomial (incl. use of contaminated needles) | 21wk | 4 towns | NR |
| 1979  South Sudan^d^ | 34 total  10 confirmed | 22  65% (46-80%) | Spillover, bats suspected | Textile factory worker | Intrafamilial, nosocomial | 10wk | 2 towns | 38% M  Age range 10m-60y  6% <19y  6% HCW |
| 2000-01  Uganda | 425 total  218 confirmed | 224  53% (48-58%) | Spillover | Farmer | Funeral, intrafamilial, nosocomial | 24wk | 3 districts | 37% M  Age range 3d-72y  Median age 27y  7% HCW |
| 2004  South Sudan^d^ | 17 total  13 confirmed | 7  41% (18-67%) | Single spillover from NHP | Villager with recent hunting history | Intrafamilial, nosocomial | 10wk | 1 town | 59% M  Age range 6m-60y  Median age 33y  6% <15y |
| 2011  Uganda | 1 confirmed | 1  100% (3-100%) | Single spillover, bats suspected | Child from rural village | None | NA | - | F  12y |
| 2012  Uganda | 24 total  11 confirmed | 17  71% (49-87%) | Spillover | Villager with recent history of agricultural work | Funeral, intrafamilial, nosocomial | 5wk | 1 town | NR |
| 2012  Uganda | 7 total  6 confirmed | 4  57% (18-90%) | Spillover | Motorcycle taxi rider | Funeral, intrafamilial | 6wk | 1 town | NR |
| 2022  Uganda | 164 total  142 confirmed | 77  47% (39-55%) | Spillover, bats suspected | Villager | Funeral, household, nosocomial, sexual, vertical | 16wk | 9 districts | 58% M  Median age 29y  IQR 20-38y  13% HCW |
| 2025  Uganda | 14 total  12 confirmed | 4  29% (8-58%) | Single spillover | HCW | Community, intrafamilial, nosocomial, vertical | 5wk | 6 districts | 55% M  Age range 20d-55y  Mean age 27y  50% HCW |

^a^Total cases include laboratory confirmed and probable cases. Numbers of laboratory-confirmed cases are presented alongside total cases where available. ^b^Exact (Clopper-Pearson) 95% CI were calculated, assuming a binomial distribution. ^c^Approximate estimate of duration, including the likely first cases before the outbreak was confirmed, but excluding the 42-day countdown to declare the end of the outbreak. ^d^At the time known as Southern Sudan, within Sudan. Abbreviations: CFR, case fatality rate; CI, confidence interval; d, days; F, female; HCW, healthcare workers; IQR, interquartile range; M, male; m, months; N, number; NA, not applicable; NHP, non-human primate(s); NR, not reported; SVD, Sudan virus disease; wk, weeks; y, years.

**Table 3.** **Characteristics of EVD outbreaks and epidemics reported in sub-Saharan Africa to date.** Epidemiologically linked outbreaks spanning multiple countries are grouped and summarized jointly.

| **Year, Location** | **N cases^a^** | **N deaths**  **CFR (95% CI)^b^** | **Source** | **Index case(s)** | **Human-to human transmission** | **Outbreak duration^c^** | **Geographic spread** | **Case demographics** |
| --- | --- | --- | --- | --- | --- | --- | --- | --- |
| 1976  DRC | 318 total | 280  88% (84-91%) | Spillover from NHP, antelope | Hospital patient | Community, household, nosocomial (incl. use of contaminated needles) | 7wk | 55 villages | 44% M  20% <15y  5% HCW |
| 1977  DRC | 1 total | 1  100% (3-100%) | Suspected resurgence from 1976 outbreak | Child | None | NA | NA | F  9y |
| 1994-95  Gabon | 51 total | 31  61% (46-74%) | Spillover, NHP suspected | Gold Miners | Community, household, nosocomial | 6wk | 10 villages | NR |
| 1995  DRC | 315 total | 254  81% (76-85%) | Spillover | Charcoal worker & farmer | Community, household, nosocomial | 30wk | ~30 villages | 20–25% HCW |
| 1996  Gabon | 31 total | 21  68% (49-83%) | Spillover from NHP | Hunters | Household | 12wk | 2 villages | 58% children |
| 1996-97  Gabon & South Africa | 62 total | 46  74% (62-84%) | Spillover from NHP, importation | Hunter | Community, nosocomial | 26wk | 2 countries; 3 regions in Gabon | NR |
| 2001-02  ROC & Gabon | 124 total  37 confirmed | 97  78% (70-85%) | Multiple spillovers from NHP, antelope, Importation | Hunters | Community, nosocomial | 20wk | 5 districts across 2 countries | 50% M  Age range 0-85y  27% <15y  2% HCW |
| 2002-03  ROC | 143 total | 128  90% (83-94%) | Spillover from NHP, antelope | Hunters | Community, household | 19wk | 2 districts | 53% M  Age range: 5d – 80y |
| 2003  ROC | 35 total | 29  83% (66-93%) | Spillover from NHP or boar | Hunters | Community, household | 4wk | 1 district | NR |
| 2005  ROC | 12 total | 10  83% (52-98%) | Multiple spillovers from NHP, antelope | Poachers | Community, household | 5wk | 2 districts | 83% M  Age range 16-57y |
| 2007  DRC | 264 total | 187  71% (65-76%) | Spillover, bats suspected | Village Chief & hunter | Community, nosocomial | 9wk | 2 health zones | NR |
| 2008  DRC | 32 total | 15  47% (29-65%) | Suspected resurgence from 2007 outbreak | Postpartum mother | Community, nosocomial | 11wk | 1 district | NR |
| 2013-16  W Africa | 28,639 total  28,610 confirmed | 11,308  39% (39-40%) | Spillover, bats suspected; Importation | Infant | Community, household, funeral, nosocomial transmission, lab-acquired | 28m | 6 countries in SSA, & 10 countries worldwide | 5% HCW |
| 2014  DRC | 69 total  66 confirmed | 49  71% (59-81%) | Spillover from NHP | Pregnant woman, married to bushmeat hunter | Funeral, community | 12wk | 4 districts | 52% F  Age range <5-60y  12% HCW |
| 2017  DRC | 8 total | 4  50% (16-84%) | Single spillover from NHP, wild boar | Villager with recent history of butchering bushmeat | Community, nosocomial | 3wk | 7 towns | 75% M  Age range 16-60y  13% <18y |
| 2018  DRC | 54 total  38 confirmed | 33  61% (47-74%) | Spillover | Police officer | Community, nosocomial | 11wk | 3 health zones | 60% M  Age range 8-80y  Median 41y  21% HCWs |
| 2018-20  DRC^b^ | 3,470 total  3,317 confirmed | 2,287  66% (64-67%) | Spillover | Villagers with history of bush meat consumption | Community, household, nosocomial, funeral | 23m | 18 health zones | 56% F  Age range: 0-80y  29% <18 years  5% HCWs |
| 2020  DRC | 130 total  119 confirmed | 55  42% (34-51%) | Spillover & suspected resurgence from 2018 outbreak | Woman with frequent bat consumption | Community, funeral, household, nosocomial | 20wk | 13 health zones | 55% M  Age range: <5-55+  23% <18y  2% HCW |
| 2021  DRC | 12 total  11 confirmed | 6  50% (21-79%) | Suspected resurgence from 2018-20 outbreak | Suspected 2018-2020 survivor | Household, nosocomial, possible sexual transmission | 12wk | 4 districts | 17% HCW |
| 2021  DRC | 11 total  8 confirmed | 9  82% (48-98%) | Suspected resurgence from 2018-20 outbreak | 3-year-old child | Community, nosocomial | 10wk | 4 towns | 50% <5y |
| 2021  Guinea | 23 total  16 confirmed | 12  52% (31-73%) | Suspected resurgence from 2013-16 epidemic | HCW | Funeral, nosocomial | 18wk | 2 regions | 22% HCW |
| 2022  DRC | 5 total  4 confirmed | 5  100% (48-100%) | Spillover | Student | Community, intrafamilial | 10wk | 3 towns | 80% M  Age range 9-48y |
| 2022  DRC | 1 total  1 confirmed | 1  100% (3-100%) | Suspected resurgence from 2018-20 outbreak | Hospitalized patient | None | NA | NA | F  46y |
| 2025  DRC | 64 total  53 confirmed | 45  70% (58-81%) | Single spillover | Pregnant woman | Community, funeral, household, nosocomial, vertical | 6wk | 1 health zone | 42% M  Age range 0-65y  25% <9y  8% HCW |

^a^Total cases include laboratory confirmed and probable cases. Numbers of laboratory-confirmed cases are presented alongside total cases where available. ^b^Exact (Clopper-Pearson) 95% CI were calculated, assuming a binomial distribution. ^c^Approximate estimate of duration, including the likely first cases before the outbreak was confirmed, but excluding the 42-day countdown to declare the end of the outbreak. ^d^Four EVD cases from the 2019 DRC outbreak were recorded in both DRC and Uganda due to cross-border movement; all four cases acquired infection and died in the DRC, so Uganda is omitted for the purpose of this table. Abbreviations: CFR, case fatality rate; CI, confidence interval; d, days; DRC, Democratic Republic of Congo; EVD, Ebola virus disease; F, female; HCW, healthcare workers; IQR, interquartile range; M, male; m, months; N, number; NA, not applicable; NHP, non-human primate(s); NR, not reported; ROC, Republic of Congo; SSA, Sub-Saharan Africa; W Africa, West Africa; wk, weeks; y, years.

**
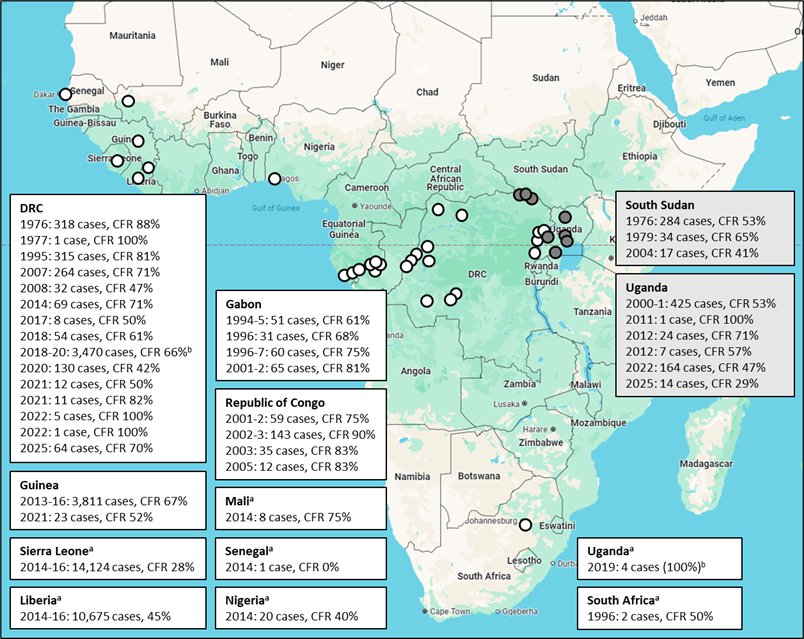
**

**Figure 1. History of reported EVD and SVD outbreaks in sub-Saharan Africa, by country.** EVD outbreaks are shown by white dots, and brief details (year of outbreak, number of reported cases, CFR) are provided in white boxes. SVD outbreaks are shown by grey dots, and brief details are provided in grey boxes. ^a^EVD outbreaks in these countries resulted from cross-border spread of infection from another affected country. ^b^Four EVD cases from 2019 were recorded in both the DRC and Uganda due to cross-border movement; all four cases acquired infection and died in the DRC. Abbreviations: CFR, case-fatality rate; DRC, Democratic Republic of Congo; EVD, Ebola virus disease; SVD, Sudan virus disease.

**
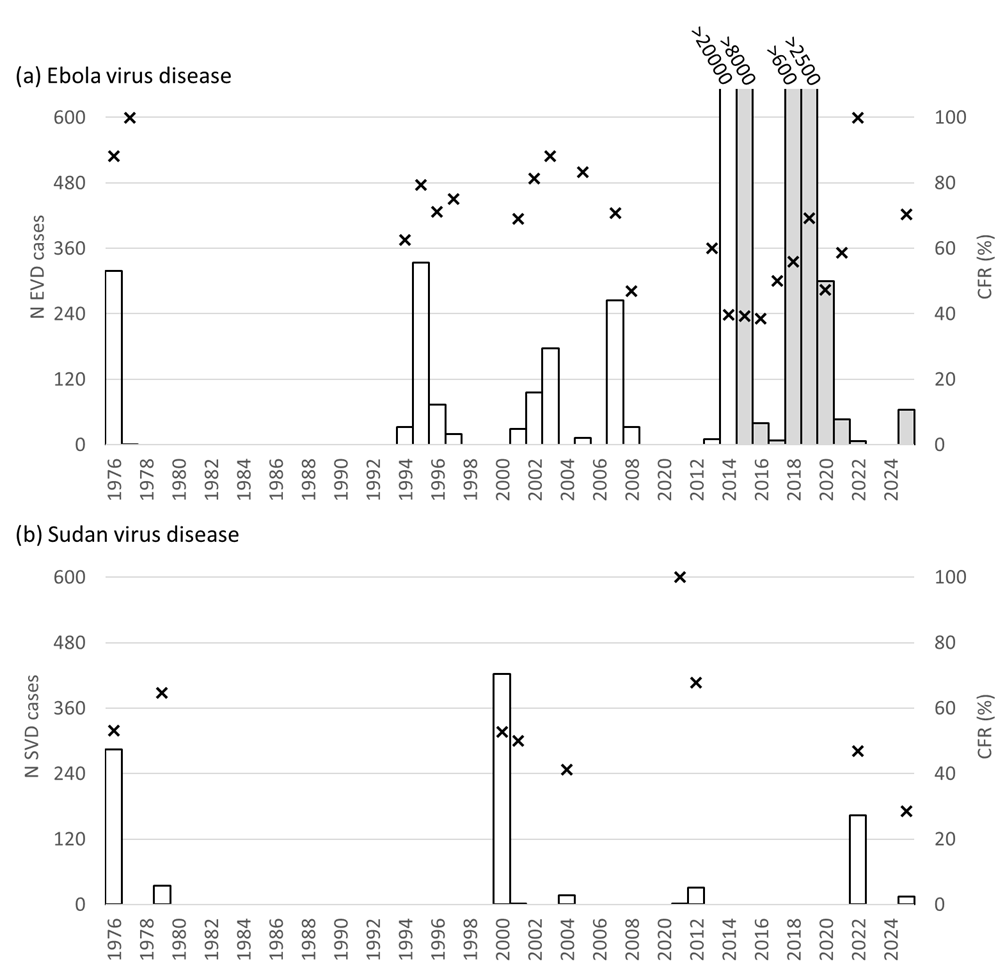
**

**Figure 2. Number of reported disease cases (bars) caused by (a) EBOV and (b) SUDV and associated CFRs (crosses) per year since 1976.** For multi-year outbreaks, annual distributions of cases and CFRs were approximated using data from periodic outbreak updates. In instances where multiple outbreaks occurred in the same year, figures reflect cumulative case counts and combined CFRs. Grey bars are shown for years when EBOV vaccines were available during EVD outbreaks, either in a trial setting or through wider vaccine deployment. Abbreviations: CFR, case-fatality rate; EBOV, Ebola virus; EVD, Ebola virus disease; N, number; SUDV, Sudan virus; SVD, Sudan virus disease.


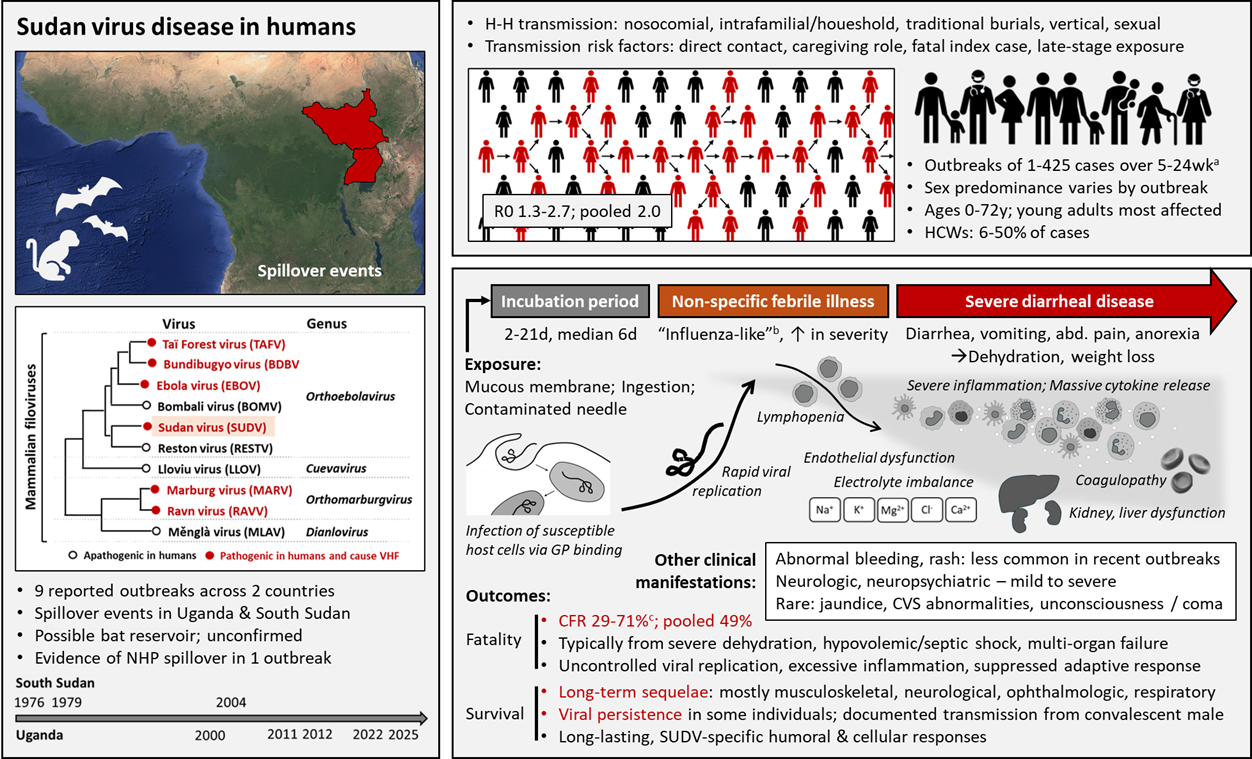


**Figure 3. Summary of Sudan virus disease (SVD) in humans.** The map in the left panel shows countries in which Sudan virus (SUDV) spillover events and associated SVD outbreaks have been reported. The timeline at the bottom of the same panel indicates the years in which SVD outbreaks were reported in South Sudan (formerly Southern Sudan) and Uganda; the outbreak that was reported in 2000 continued into 2001, and two outbreaks occurred in 2012. The phylogenetic tree was adapted from Groseth and Hoenen (NPJ Viruses, 2024). The graphic in the lower right panel depicts the current understanding of the routes and progression of SUDV infection and disease in humans. This is intended as an approximate representation and does not reflect exact timings, highlighting uncertainty in the existing evidence on human disease. ^a^Approximate estimates of duration, including the likely first cases before the outbreaks were confirmed, but excluding the 42-day countdown to declare the end of an outbreak. ^b^Including headache, fatigue, weakness, malaise, myalgia, arthralgia, anorexia, cough, and chest pain. ^c^Excluding the 2011 outbreak that consisted of just a single fatal case. Abbreviations: abd., abdominal; CFR, case fatality rate; CVS, cardiovascular system; d, days; GP, glycoprotein; HCWs, healthcare workers; H-H, human-to-human; NHP, non-human primates; R0, basic reproduction number; VHF, viral hemorrhagic fever; wk, weeks; y, years.


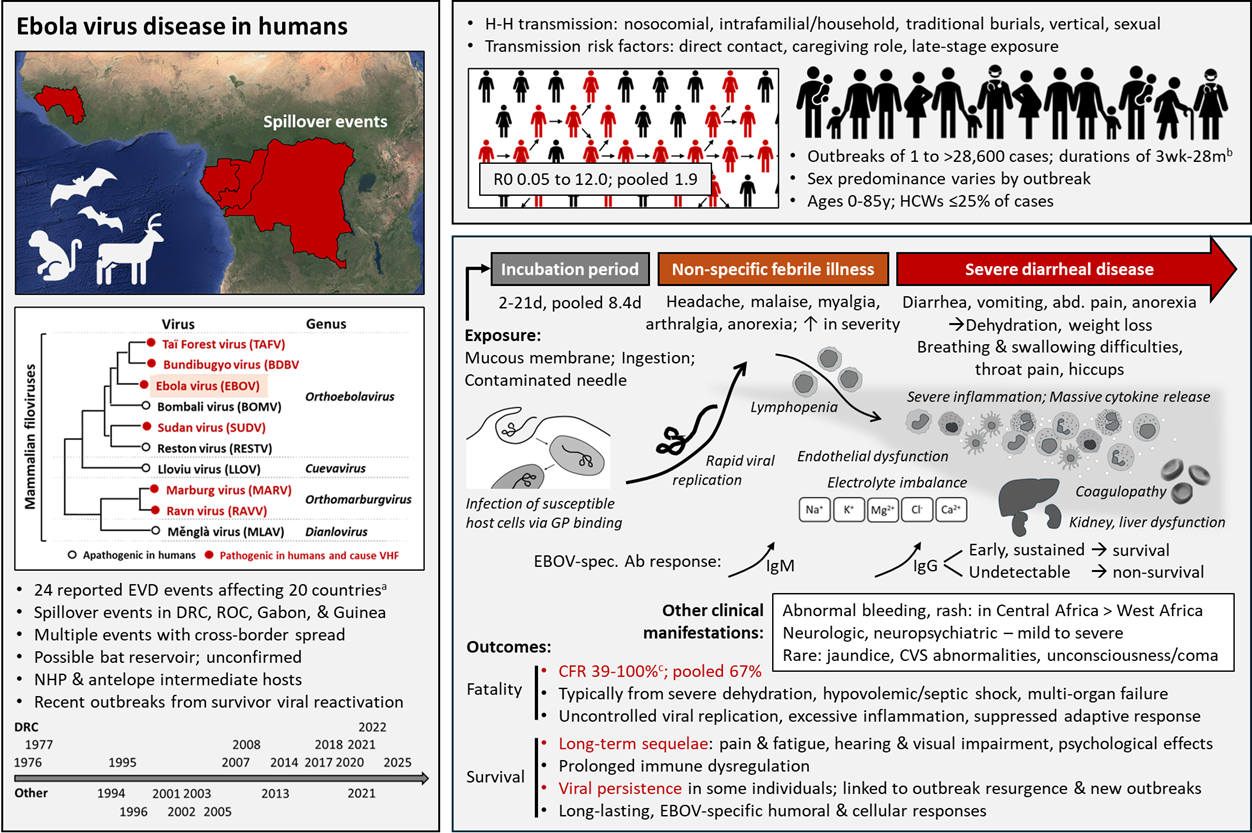


**Figure 4. Summary of Ebola virus disease (EVD) in humans.** The map in the left panel shows countries in which Ebola virus (EBOV) spillover events have been reported. Owing to cross‑border spread during several outbreaks, the number of countries affected by EVD outbreaks is substantially larger than the number of primary spillover locations. The timeline at the bottom of the same panel indicates the years in which EVD outbreaks were reported in the Democratic Republic of the Congo (DRC), where most EVD outbreaks have occurred to date, and in other countries; some outbreaks spanned multiple years, and some years saw multiple outbreaks. The phylogenetic tree was adapted from Groseth and Hoenen (NPJ Viruses, 2024). The graphic in the lower right panel depicts the current understanding of the routes and progression of EBOV infection and disease in humans. This schematic is intended as an approximate representation and does not reflect exact timings, highlighting uncertainty in the existing evidence on human disease. ^a^The 24 events include epidemiologically linked outbreaks grouped across countries; disaggregation by country yields 32 outbreaks in SSA. The 20 affected countries include four in sub‑Saharan Africa (SSA) with documented zoonotic spillover, seven additional SSA countries affected through cross‑border transmission, and nine countries outside SSA affected via importation (including medical evacuation). ^b^Approximate estimates of duration, including the likely first cases before the outbreaks were confirmed, but excluding the 42-day countdown to declare the end of an outbreak. Abbreviations: Ab, antibody; abd., abdominal; CFR, case fatality rate; CVS, cardiovascular system; d, days; GP, glycoprotein; HCWs, healthcare workers; H-H, human-to-human; IgG, Immunoglobulin G; IgM, Immunoglobulin M; m, months; NHP, non-human primates; R0, basic reproduction number; ROC, Republic of Congo; VHF, viral hemorrhagic fever; wk, weeks; y, years.
